# Low-Intensity Extracorporeal Shock Wave Therapy Promotes Bladder Regeneration and Improves Overactive Bladder Induced by Ovarian Hormone Deficiency from Rat Animal Model to Human Clinical Trial

**DOI:** 10.3390/ijms22179296

**Published:** 2021-08-27

**Authors:** Kun-Ling Lin, Jian-He Lu, Kuang-Shun Chueh, Tai-Jui Juan, Bin-Nan Wu, Shu-Mien Chuang, Yung-Chin Lee, Mei-Chen Shen, Cheng-Yu Long, Yung-Shun Juan

**Affiliations:** 1 Graduate Institute of Clinical Medicine, College of Medicine, Kaohsiung Medical University, Kaohsiung 80708, Taiwan; nancylin95@gmail.com (K.-L.L.); spacejason69@yahoo.com.tw (K.-S.C.); 2 Department of Obstetrics and Gynecology, Kaohsiung Municipal Ta-Tung Hospital, Kaohsiung 80661, Taiwan; 3 Department of Obstetrics and Gynecology, Kaohsiung Medical University Hospital, Kaohsiung 80756, Taiwan; 4 Emerging Compounds Research Center, Department of Environmental Science and Engineering, College of Engineering, National Pingtung University of Science and Technology, Pingtung 91201, Taiwan; toddherpuma@yahoo.com.tw; 5 Department of Urology, Kaohsiung Municipal Ta-Tung Hospital, Kaohsiung 80661, Taiwan; 6 Department of Urology, Kaohsiung Medical University Hospital, Kaohsiung 80756, Taiwan; 7 Department of Medicine, National Defense Medical College, Taipei 11490, Taiwan; terryjuan@gmail.com; 8 Department of Pharmacology, College of Medicine, Kaohsiung Medical University, Kaohsiung 80708, Taiwan; binnan@kmu.edu.tw; 9 Department of Urology, College of Medicine, Kaohsiung Medical University, Kaohsiung 80708, Taiwan; u9181002@gmail.com (S.-M.C.); bear5824@gmail.com (M.-C.S.); 10 Department of Urology, Kaohsiung Municipal Siaogang Hospital, Kaohsiung 81267, Taiwan; leeyc12345@yahoo.com.tw

**Keywords:** low-intensity extracorporeal shock wave therapy, ovarian hormone deficiency, overactive bladder, bladder angiogenesis

## Abstract

Postmenopausal women with ovary hormone deficiency (OHD) are subject to overactive bladder (OAB) symptoms. The present study attempted to elucidate whether low-intensity extracorporeal shock wave therapy (LiESWT) alters bladder angiogenesis, decreases inflammatory response, and ameliorates bladder hyperactivity to influence bladder function in OHD-induced OAB in human clinical trial and rat model. The ovariectomized (OVX) for 12 months Sprague–Dawley rat model mimicking the physiological condition of menopause was utilized to induce OAB and assess the potential therapeutic mechanism of LiESWT (0.12 mJ/mm^2^, 300 pulses, and 3 pulses/second). The randomized, single-blinded clinical trial was enrolled 58 participants to investigate the therapeutic efficacy of LiESWT (0.25 mJ/mm^2^, 3000 pulses, 3 pulses/second) on postmenopausal women with OAB. The results revealed that 8 weeks’ LiESWT inhibited interstitial fibrosis, promoted cell proliferation, enhanced angiogenesis protein expression, and elevated the protein phosphorylation of ErK1/2, P38, and Akt, leading to decreased urinary frequency, nocturia, urgency, urgency incontinence, and post-voided residual urine volume, but increased voided urine volume and the maximal flow rate of postmenopausal participants. In conclusion, LiESWT attenuated inflammatory responses, increased angiogenesis, and promoted proliferation and differentiation, thereby improved OAB symptoms, thereafter promoting social activity and the quality of life of postmenopausal participants.

## 1. Introduction

Postmenopausal women with ovary hormone deficiency (OHD) status could cause urological dysfunctions, including overactive bladder (OAB) symptoms, stress urinary incontinence (SUI), and recurrent urinary tract infection (UTI) [1]. Up to 45% of postmenopausal women with vaginal atrophy are associated with urogenital atrophy and urinary symptoms, including increased urinary frequency, nocturia, urgency, urgency incontinence, and recurrent UTI [2,3,4]. Epidemiological studies have shown that estrogen deficiency is associated with an increased risk of lower urinary tract symptoms (LUTSs) in ageing women [5]. The clinically efficacy of estrogen replacement for bladder dysfunction (e.g., OAB) during and after the menopause has been investigated [6]. However, postmenopausal women with estrogen deficiency are usually reluctant to estrogen therapy due to fear of hormone related cancer: such as endometrial or breast malignancy [7]. More than 60% of postmenopausal women refused hormone therapy in the two years of treatment due to the above cancerphobia [8].

Ovarian hormone, especially 17ß estradiol plays a key role in regulation of bladder growth, differentiation, and function [9]. Estrogen application improved urothelial proliferation and differentiation via estrogen receptor**-**α (ER**-**α) and estrogen receptor**-**ß (ER**-**ß) receptors [10]. In ER-ß**-**/**-**female mice, ulceration and atrophy of urothelium were displayed compatible with clinical interstitial cystitis/bladder pain syndrome (IC/BPS) [11]. Estrogen supplement can improve OAB symptoms and urinary incontinence among postmenopausal women [12]. As is well known, the ovariectomized (OVX) rat model can mimic the physiological condition of OHD and induce OAB [8,13,14]. Previous studies revealed that the estrogen**-**depletion rats had damaged bladder UL and changed the prostaglandin level [15]. Additionally, OVX rats had bladder smooth muscle atrophy, decreased muscle contractility, enhanced mucosal atrophy, diminished blood flow, and finally resulted in tissue hypoxia [16,17]. Similarly, Lin et al. investigated the influence of OVX in rabbits and found estrogen deficiency diminished bladder contractility and changed regulatory proteins, and estrogen supplementation improved bladder overactivity [18]. Our previous study also showed OVX rats resulted in diminishing bladder compliance, increasing oxidative damage, enhancing interstitial fibrosis, and augmenting bladder mucosa apoptosis [19]. The reason why estrogen therapy can improve LUTSs might be via increasing urogenital blood flow [13,14]. Estradiol administration also improved OVX-induced vascular degeneration and vascular density reduction by increased vascular density within detrusor smooth muscle bundles [20,21].

The International Continence Society (ICS) defined OAB syndrome as urinary frequency and nocturia accompanied by urgency with or without urgency incontinence, without evidence of urinary tract infection or other pathological process [22,23]. According to previous reports, about 35% of postmenopausal women aged over 65 years old were disturbed by OAB [3]. In Asian countries, a questionnaire**-**based survey performed in 5502 females showed the prevalence of female OAB was 53.1% [24]. The symptom of OAB has a significant negative affect on the quality of life (QoL) and social activities. Moreover, increased nocturia frequency may cause depression and fatigue. In particular, urgency incontinence was associated with an increase in falls and fractures incidence of the elderly population [25]. However, the pathophysiology of OAB was composed of multiple possible causes which need further elucidated.

The guideline for treatment of OAB includes behavior modification, pelvic floor muscle exercise, estrogen treatment, and drug administration [9]. Clinically, the pharmacotherapies of OAB in postmenopausal women have two classes of medications: anti**-**muscarinic and beta**-**adrenergic agents. However, adverse effects with dry mouth, constipation, and unstable blood pressure control make OAB women unwilling to continue oral medications. Besides, local vaginal estrogen therapy has been shown to relieve OAB symptoms, reduce urinary incontinence, and improve the QoL [26]. Bladder Botox injection and tibial nerve stimulation were the second line treatment due to Invasive treatment with side effects: increased residual urine, catheter drainage risk, hematuria, and neural pain. Therefore, a non**-**drug and non**-**invasive treatment for management of postmenopausal OAB is pursued in recent decades. In human clinical trial, LiESWT has been used widely for various types of diseases, including chronic pelvic pain syndrome (CPPS) [27,28,29,30,31], erectile dysfunction (ED) [32,33,34,35,36], myocardial infarction (MI) [37], OAB [38], stress urinary incontinence (SUI) [39], and IC/BPS. Zimmermann et al. investigated the effect of LiESWT (0.10–0.25 mJ/mm^2^, 3000 impulses for 4 weeks) on non-bacterial prostatitis/CPPS and showed improvement in perineal pain and LUTS, as well as erectile function and QoL [27]. Besides, evidence suggested that LiESWT treatment could induce penile tissue regeneration and increased penile hemodynamics for ED patients [32]. Besides, 4 weeks of LiESWT (2000 shocks, frequency of 3 Hz, energy of 0.25 mJ/mm^2^, and once a week) at suprapubic area can improve urinary frequency, bladder inflammation, and pain with a significant decrease in VAS pain scale in refractory IC/BPS patients [40]. Additionally, our previous results suggested that 8**-**week LiESWT (0.25 mJ/mm^2^, 3000 pulses, 3 Hz, and once a week) attenuated SUI symptoms on physical activity, reduced bladder leaks, and improved in the QoL [39]. Our other study on OAB also showed that 8**-**week LiESWT (0.25 mJ/mm^2^, 3000 pulses, and 3 Hz) could ameliorate OAB symptoms and increase voided urine volume, maximum urinary flow rate (Qmax), decrease postvoid residual urinary volume (PVR), and improve functional bladder capacity [38]. Importantly, the advantages of LiESWT include outpatient-based, short treatment sessions, with no anesthesia required and without medication or surgery.

In rat model with cyclophosphamide (CYP) induced-IC, LiESWT improved bladder overactivity through inducing angiogenesis and reducing oxidative stress [41]. Chen et al. showed that LiESWT noticeably alleviated bladder damage and reduced oxidative stress by decreasing IL**-**12, MMP9, TNF**-**α, NF**-**κB, and iNOS expression in rats with CYP**-**induced IC [41]. Additionally, Wang et al. reported that 4**-**week LiESWT improved streptozotocin-induced diabetic rat ameliorated diabetic bladder dysfunction and decreased urinary incontinence by ameliorated bladder wall composition, enhanced detrusor contractility of bladder and urethra, nerve innervation and muscle regeneration of bladder, and promoted urethra continence [42]. However, the molecular mechanism of the effect of LiESWT on OAB is still not clear.

This study provided both OAB animal model and human clinical trial to investigate the therapeutic efficacy of LiESWT and its underlying mechanisms. In animal models, the OVX rat model mimicking the physiological condition of menopause was utilized to induce OAB. We hypothesized that LiESWT could ameliorate detrusor overactivity by attenuating the inflammatory responses, increasing angiogenesis, and strengthening cell proliferation to improve OAB symptoms. In the clinical trial, we explored the clinical application of LiESWT on postmenopausal OAB women and its therapeutic efficacy, including ameliorating OAB symptoms, increasing voided volume, and improving social activity and QoL.

## 2. Results

### 2.1. Part I: OAB Animal Model Experiment

#### 2.1.1. Serum Estradiol Concentration Reduced after Bilateral Ovariectomy (OVX)

Surgical OVX in rat models was applied to mimic women’s menopausal status with OHD. As seen in Table 1, the concentration of serum estradiol was significantly decreased after two weeks of OVX surgery. The levels declined from 32.33 ± 1.52 (baseline data) to 16.53 ± 1.28 pg/mL in the OVX group, from 31.75 ± 1.29 to 15.59 ± 0.99 pg/mL in the OVX + SW4 group, and from 32.35 ± 0.53 to 15.65 ± 1.36 pg/mL in the OVX + SW8 group in comparison with the sham group: from 32.17 ± 1.41 to 33.64 ± 3.66 pg/mL. According to the above data, OVX surgery really resulted in estradiol deficiency.

#### 2.1.2. Physical Characteristics

The physical indicators are shown in Table 1, including water intake, urine output, body weight, bladder weight, and the ratio of bladder weight and body weight. There was no significant difference in the amount of water intake and urine output among different groups. In addition, the body weight increased noticeably in groups of OVX, OVX + SW4, and OVX + SW8 as compared to the sham group (*p* < 0.01). Furthermore, bladder weight of OVX + SW8 (205.00 ± 25.88 mg) was significantly increased compared to the OVX group (163.33 ± 10.33 mg). Especially, the ratio of bladder weight and body weight significantly lowered in the OVX group (0.32 ± 0.02) and the OVX + SW4 group (0.33 ± 0.10) compared with the sham group (0.46 ± 0.07).

#### 2.1.3. LiESWT Treatment Ameliorated Bladder Overactivity

**Figure 1:**
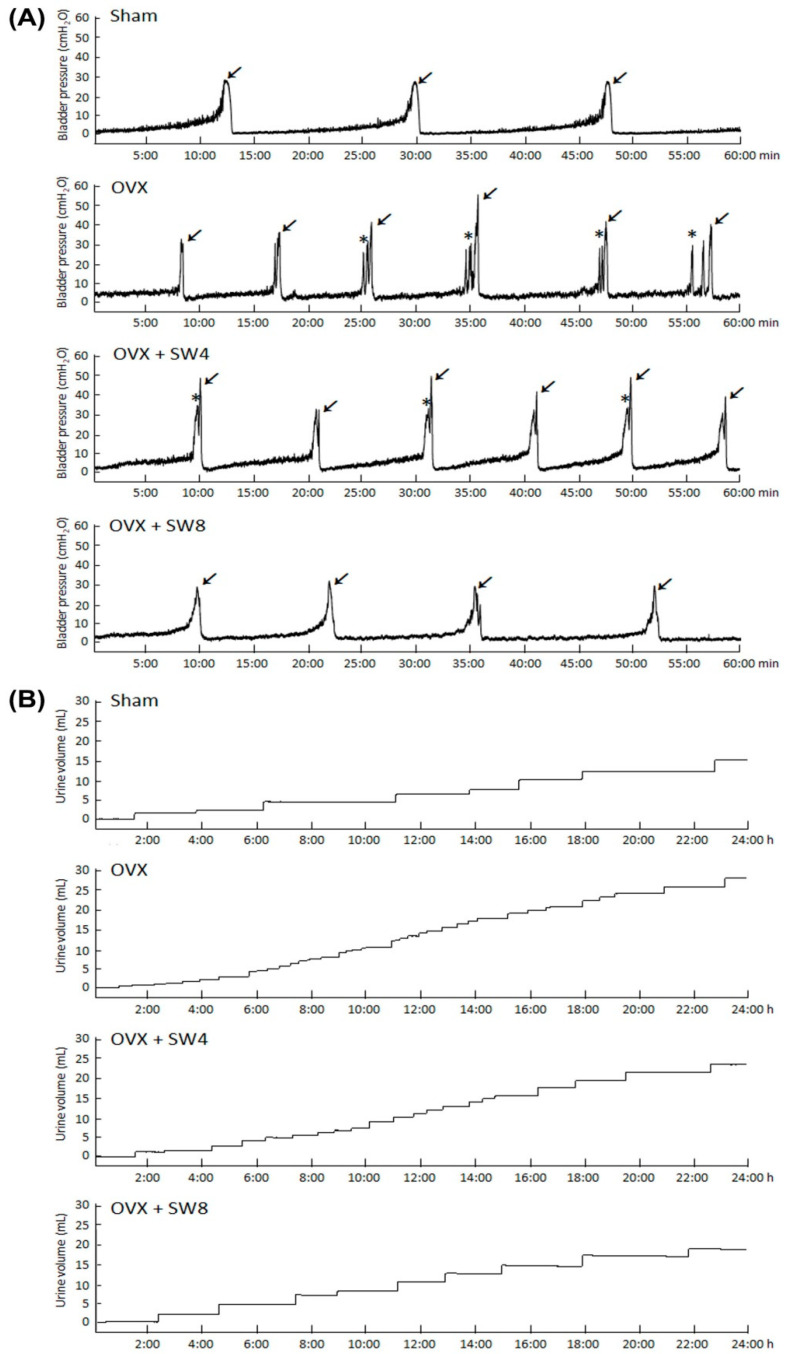
Urodynamic analysis of bladder cystometric parameters and voiding behavior shown in an OVX induced OAB of rat model. (**A**) Cystometry recordings of micturition pressure, voiding volume, and frequency, including voiding contraction (arrows) and non-voiding contraction (asterisks). (**B**) Tracing analysis of 24-h voiding behavior by metabolic cage. The OVX group significantly increased bladder maturation pressure, voiding contraction, non-voiding contraction, and micturition frequency, whereas LiESWT treatment significantly improved bladder voiding pattern and capacity.

The bladder function was analyzed by urodynamic parameters and voiding behavior, including peak micturition pressure, micturition frequency, voided volume, and non-voided contraction, and the data are shown in Table 1 and Figure 1. The sham group showed a regular and stable micturation pattern. However, the OVX group revealed bladder hyperactivity with increased micturition frequency (arrows), peak micturition pressure and non-voiding contraction (asterisks) as compared with the sham group. In contrast, both the OVX + SW4 group and the OVX + SW8 group significantly reduced peak micturition pressure as well as micturition frequency, and augmented bladder capacity as compared with the OVX group (Figure 1A and Table 1).

Additionally, tracing analysis of voiding behavior by metabolic cage revealed that the OVX group decreased voiding volume and increased micturition frequency as compared with the sham group (Figure 1B). In the OVX + SW8 group, there was an increase in the micturation interval and the voiding volume as compared with those in the OVX group. Therefore, the bladder storage function was decreased in the OVX group, whereas it was significantly improved after LiESWT treatment, both in the 4-week and 8-week groups. Taken together, the above findings revealed that OVX treatment deteriorated bladder capacity and exacerbated storage function, whereas LiESWT treatment significantly improved bladder capacity and ameliorated overactive bladder induced by OVX.

#### 2.1.4. Effects of LiESWT on OVX-Induced Bladder Interstitial Fibrosis

**Figure 2:**
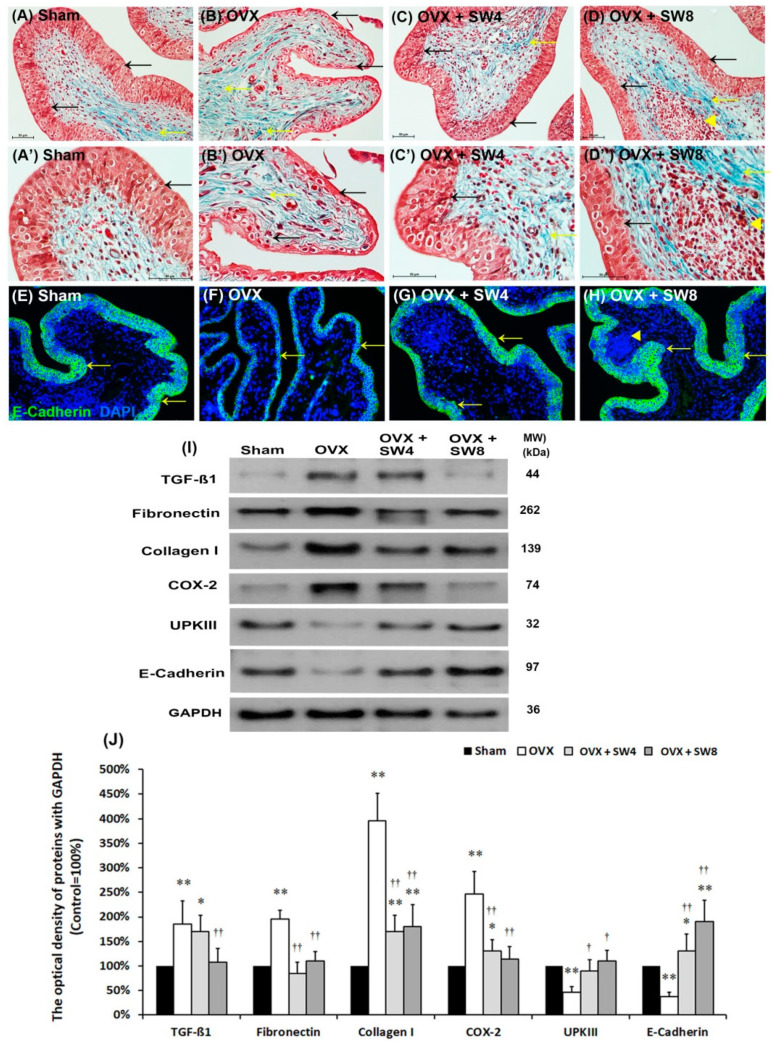
Histopathological examination for bladder damage was shown by Masson’s trichrome staining, immunostaining, and Western blots. (**A**–**D**, **A′**–**D′**) The bladder pathological features of the sham group (**A**,**A′**), the OVX group (**B**,**B′**), the OVX + SW4 group (**C**,**C′**), and the OVX + SW8 group (**D**,**D′**). Masson’s trichrome stain showed the navy blue-stained nucleus, green-stained collagen, and red-stained smooth muscle were highlighted. In the sham group (**A**,**A′**), there were three to five layers of UL and only sparse collagen (yellow arrow) distributed in the SL. In the OVX group (**B**,**B′**), the morphology was characterized by thinner layer of UL (black arrows), and much collagen accumulation and interstitial fibrosis in the SL (yellow arrows). In contrast, the pathological features of the OVX + SW4 group (**C**,**C′**) and the OVX + SW8 group (**D**,**D′**) showed improved OVX-associated bladder damages by increasing a thicker layer of urothelium (black arrows) and reducing interstitial fibrosis (yellow arrows) compared with the OVX group. Particularly, there are many mononuclear cell groups gathered into sphere (yellow arrowheads) and much collagen accumulation (yellow arrows) around the sphere in the SL of the OVX + SW8 group (**D**,**D′**). (**E**–**H**) The distribution of cell-adhesion marker E-cadherin by immunostaining was shown. In the sham group (**E**), the E-cadherin staining was found in intercellular junctions of urothelium. On the contrary, there was less E-cadherin staining expression in the thin urothelium of the OVX group (**F**), but the immunostaining of the OVX + SW4 group (**G**) and the OVX + SW8 group (**H**) was enhanced the staining in the UL. (**I**,**J**) Western blot was evaluated the protein levels of bladder inflammation (TGF**-**ß1 and COX**-**2), interstitial fibrosis (fibronectin and type I collagen), and urothelial structure (E-cadherin and uroplakin III). Both the inflammatory and fibrosis markers (TGF**-**ß1, fibronectin, type I collagen, and COX**-**2) were significantly increased in the OVX group compared to the sham group. However, the proteins noticeably decreased in the OVX + SW4 group and the OVX + SW8 group compared to the OVX group except TGF**-**ß1 expression in the OVX + SW4 group. Note: CK, cytokeratin; UL, urothelial layer; UBL, urothelial basal layer; SL, suburothelial layer; UPKIII, uroplakin III; TGF**-**ß1, transforming growth factor ß 1. Data were expressed as mean ± SD for *n* = 6, * *p* < 0.05; ** *p* < 0.01 versus the sham group. ^†^ *p* < 0.05; ^††^ *p* < 0.01 versus the OVX group.

The difference in the bladder tissue marked by Masson’s trichrome stain observed among groups is shown in Figure 2. In comparison with the sham group (Figure 2A,A′), the morphology of the OVX group was characterized by a thinner layer of urothelial cells (black arrows), damaged mucosal integrity, and much collagen accumulation (yellow arrows) in the suburothelial layer (SL; lamina propria) (Figure 2B,B′). Additionally, morphological evaluation of the OVX + SW4 group and the OVX + SW8 group showed improved OVX-associated bladder damages by increasing a thicker layer of urothelium (black arrows) and reducing interstitial fibrosis (yellow arrows) compared with the OVX group (Figure 2C,C′,D,D′). In particular, there were many mononuclear cell groups gathered into the sphere (yellow arrowheads) and much collagen accumulation (yellow arrows) around the sphere in the SL of the OVX + SW8 group (Figure 2D,D′). 

We investigated the distribution of the cell-adhesion marker E-cadherin as urothelial barrier function indicator in the bladder tissue (Figure 2E–H). As shown in Figure 2E of the sham group, the E-cadherin staining was found in intercellular junctions of urothelium, but the suburothelial area showed no immunoreactivity of E-cadherin. On the contrary, there was less E-cadherin staining expression in the thin urothelium of the OVX group (Figure 2F), but the immunostaining of the OVX + SW4 group was enhanced (Figure 2G). The E-cadherin immunostaining in the OVX + SW8 was recovered to the sham group level (Figure 2H).

We further evaluated the protein levels of pro-inflammatory markers (TGF-ß1 and COX-2), interstitial fibrosis markers (fibronectin and type I collagen), and urothelial structure (E-cadherin and uroplakin III) by Western blotting (Figure 2I,J). Both the inflammatory and fibrosis markers (TGF-ß1, fibronectin, type I collagen, and COX-2) were significantly increased in the OVX group compared to the sham group. However, all four markers noticeably decreased in the OVX + SW4 group and the OVX + SW8 group compared to the OVX group, except for TGF-ß1 expression in the OVX + SW4 group. These observations implied that LiESWT for 4 weeks or 8 weeks reduced inflammatory reaction and inhibited interstitial fibrotic progression. Besides, the urothelial expressions of cell-adhesion marker E-cadherin and differentiation marker UPKIII in umbrella cells were significantly decreased in the OVX group compared with the sham group. Otherwise, the UPKIII marker in the OVX + SW4 group and the OVX + SW8 group was noticeably increased compared with the OVX group (Figure 2I,J). These results revealed that OVX decreased the expression of urothelial structure associated markers, at last resulted in urothelial atrophy. Based on the morphological evaluation and Western blotting results, these observations suggested that the OVX significantly exacerbated bladder pathological damage and interstitial fibrosis. On the contrary, LiESWT treatment reduced the fibrotic biosynthesis and ameliorated the bladder damage.

#### 2.1.5. LiESWT Improved OVX-Induced Pathological Alteration

**Figure 3:**
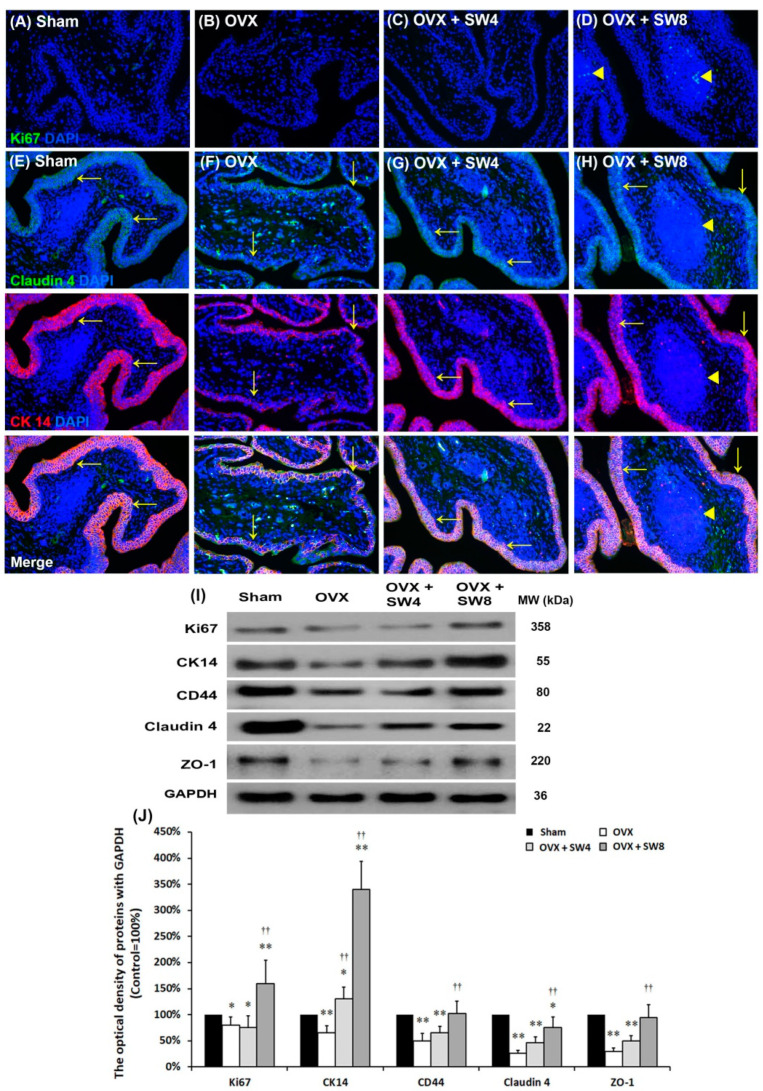
LiESWT improved OVX-induced pathological alteration and urothelial junction-associated protein expression. The expressions of proliferating and differential markers (Ki67, CK14 and CD44) and urothelial tight junction proteins (Claudin**-**4 and ZO**-**1) were assessed by immunofluorescence evaluation (**A**–**H**) and Western blot (**I**,**J**). (**A**–**D**) The staining of proliferation marker Ki67 was less distribution in the bladder tissues of the sham group (**A**), the OVX group (**B**) and the OVX + SW4 group (**C**). On the contrary, the Ki67 immunostaining was obviously expressed in the UBL and the sphere of SL in the OVX + SW8 group (**D**). (**E**–**H**) Double-labeled analysis of Claudin**-**4 (fluorescein isothiocyanate; green, upper panels) and CK14 (rhodamine; red, lower panels) was widely distributed in the UL of the sham group (**E**). The co-staining was restricted to the thin and disrupted urothelium in OVX group (**F**). However, the labeling of the OVX + SW8 group (**H**) was markedly expressed in the UBL and the sphere of SL compared to the OVX group (**F**) and the OVX + SW4 group (**G**). Nuclear DNA was labeled with DAPI (blue). (**I**, **J**) The protein levels of Ki67, CK14, CD44, Claudin**-**4, and ZO**-**1 were investigated by Western blotting analysis. The protein levels noticeably enhanced in the OVX + SW8 group compared to the OVX group. Note: CK, cytokeratin; UL, urothelial layer; UBL, urothelial basal layer; SL, suburothelial layer. Values are means ± SD for *n* = 6. * *p* < 0.05; ** *p* < 0.01 versus the sham group. ^††^ *p* < 0.01 versus the OVX group.

To investigate the therapeutic effect of LiESWT on the OVX-induced pathological alteration, the expressions of urothelial tight junction (Claudin-4 and ZO-1) and proliferating markers (Ki67 and CK14) were assessed by immunofluorescence and Western blot (Figure 3). The staining of proliferation marker Ki67 was less distribution in the bladder tissues of the sham group, the OVX group, and the OVX + SW4 group. On the contrary, the Ki67 immunostaining was obviously expressed in the UBL and the sphere of SL in the OVX + SW8 group (Figure 3A–D). Moreover, in the sham group, the co-staining of claudin-4 and CK14 was widely distributed in the UL. However, the co-staining in the OVX group was restricted to the thin and disrupted urothelium. In particular, the labeling of the OVX + SW8 group was markedly expressed in UBL and the sphere of SL compared to the OVX group (Figure 3E–H).

Western blotting analysis was further investigated the protein levels of proliferation (Ki67, CK14, and CD44) and urothelial tight junction (Claudin-4 and ZO-1) (Figure 3I,J). Both the protein levels were noticeably decreased in the OVX group compared to the sham group. However, the protein levels obviously enhanced in the OVX + SW8 group compared to the OVX group and the OVX + SW4 group. Moreover, the expression of CD44 (HA receptor) markedly decreased in the OVX group in comparison to the sham group (Figure 3I,J). However, the CD44 expression of the OVX + SW8 group was enhanced in comparison to the OVX group. Our finding revealed that LiESWT stimulated Ki67-positive (Ki67^+^) (Figure 3A–D) and CD44-positive (CD44^+^) (data not shown)-associated fibroblasts in the SL of bladder to modulate fibroblast recruitment. These findings demonstrated that LiESWT could recruit fibroblast and increase bladder regeneration to promote tissue repair through cell proliferation, differentiation, and increasing HA expression.

#### 2.1.6. LiESWT Altered Bladder Angiogenic Remodeling

**Figure 4:**
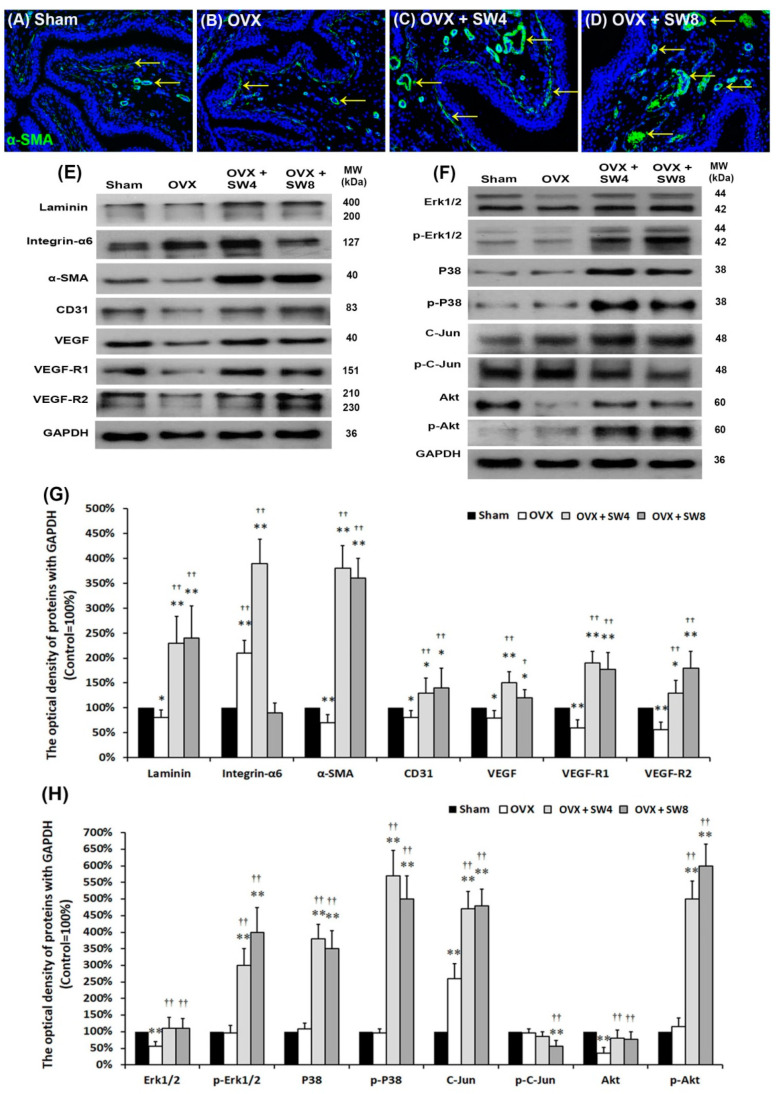
Proposed potential mechanism of regulating angiogenic remodeling triggered by LiESWT. The angiogenesis markers were analyzed by immunostaining (**A**–**D**) and Western blots (**E**–**H**). (**A**–**D**) The α**-**SMA immunostaining widely distributed in smooth muscle of microvessels beneath UBL and vessels in the SL and muscular layer in the sham group (**A**). In the OVX group (**B**), the α**-**SMA expression was reduced in the SL compared to the sham group. However, the expression levels in the OVX + SW4 group (**C**) and the OVX + SW8 group (**D**) were increased in the SL and beneath UBL. (**E**,**G**) The levels of angiogenesis-associated proteins, including Laminin, Integrin**-**α6 (Laminin receptor), α**-**SMA, CD31, VEGF, VEGF**-**R1, and VEGF**-**R2 (VEGF receptor), were quantified by Western blots. Furthermore, the protein level in the OVX group was much lower than the sham group, except Integrin**-**α6. However, the expressions of angiogenesis markers were obviously increased in the OVX + SW4 group and the OVX + SW8 group compared with the OVX group. Therefore, LiESWT altered bladder angiogenic remodeling. Note: UL, urothelial layer; UBL, urothelial basal layer; SL, suburothelial layer; VEGF, vascular endothelial growth factor. (**F**,**H**): Proposed potential mechanism involved in regulating angiogenic remodeling triggered by LiESWT. The signaling-related kinases for angiogenic response, including Erk1/2, p**-**Erk1/2, P38, p**-**P38, C**-**Jun, p**-**C**-**Jun, Akt, and p**-**Akt, were quantified by Western blots. The expression levels of both Erk1/2 and Akt proteins were significantly declined in the OVX group, but the expression level of C**-**Jun was promoted as compared with the sham group. Besides, LiESWT treatment significantly promoted the phosphorylation of Erk1/2, P38, and Akt in the bladder as compared with the OVX group, whereas it reduced the phosphorylation of C**-**Jun. Therefore, LiESWT enhanced angiogenesis through the phosphorylation of P38, Erk1/2, and Akt. Values are means ± SD for *n* = 6. * *p* < 0.05; ** *p* < 0.01 versus the sham group. ^†^ *p* < 0.05; ^††^ *p* < 0.01 versus the OVX group.

According to previous data, LiESWT might induce angiogenesis in bladder; however, the exact mechanisms remain poorly understood. To elucidate the angiogenic effect of LiESWT, the angiogenesis marker, α-SMA, was analyzed by immunostaining (Figure 4A–D). The α-SMA immunostaining was widely distributed in smooth muscle of microvessels beneath UBL and vessels in the SL (lamina propria) and ML in the sham group (Figure 4A). In the OVX group, the α-SMA immunostaining was significantly reduced compared to the sham group, while after LiESWT treatment, the α-SMA expression was significantly increased beneath UBL, both in the SL and ML in the OVX + SW4 group and the OVX + SW8 group (Figure 4A–D).

Other angiogenesis-associated proteins, including Laminin, integrin-α6, α-SMA, CD31, VEGF, VEGF-R1, and VEGF-R2, were quantified by Western blots (Figure 4E,G). This angiogenesis-associated protein expression was much lower in the OVX group than the sham group, except Integrin-α6. However, after LiESWT treatment, the expressions of these angiogenesis markers were obviously increased in the OVX + SW4 group and the OVX + SW8 group compared the OVX group. Similar expression of angiogenesis-associated protein had been observed between the OVX + SW4 group and the OVX + SW8 group (Figure 4E,G). These results showed OVX significantly decreased suburothelial and muscular vascular density, deteriorated angiogenesis, and might result in bladder tissue hypoxia and increased oxidative stress. However, LiESWT treatment improved angiogenesis, increased capillary vessels density, and VEGF expression, and thereafter increased bladder blood flow and ameliorated tissue hypoxia. Taken together, LiESWT could promote angiogenic potential and recover OVX-induced bladder hypoxia.

#### 2.1.7. Proposed Potential Mechanism for Regulating Angiogenic Remodeling Triggered by LiESWT Contributed to the Pathogenesis of OHD

In order to elucidate whether the angiogenic effect of LiESWT on OHD-induced OAB was through the VEGF/VEGFR/MAPK (Erk1/2, P38, and C-Jun) pathway or/and the Laminin/Integrin-α6/Akt pathway, the angiogenic signaling related proteins, including Erk1/2, p-Erk1/2, P38, p-P38, C-Jun, p-C-Jun, Akt, and p-Akt, were quantified by Western blots (Figure 4F,H). The expression levels of both Erk1/2 and Akt proteins were significantly declined in the OVX group, whereas the expression level of C-Jun was significantly enhanced as compared with the sham group. Besides, compared to the OVX group, the expressions of all the above proteins and phosphorylated proteins were obviously increased in the OVX + SW4 group and the OVX + SW8 group, except p-C-Jun. Our findings revealed LiESWT treatment significantly promoted the phosphorylation of Erk1/2, P38, and Akt in the bladder of the OVX + SW4 group and the OVX + SW8 group as compared with the OVX group, whereas it reduced the phosphorylation of C-Jun. Based on the above findings, we considered that LiESWT promoted bladder angiogenesis by increasing capillary density to improve OHD-induced pathological alteration, while this angiogenic enhancement was through VEGF/VEGF-R/MAPK (P38 and Erk1/2) pathways and Laminin/Integrin-α6/Akt pathways.

### 2.2. Part II: Human Clinical Trial

#### 2.2.1. Baseline Characteristics

The characteristics of 58 postmenopausal OAB participants are shown in Table 2. Physical indicators and biochemical parameters for OAB participants were evaluated. The mean age of OAB participants was 60.79 ± 1.98 years in the sham group, while the LiESWT group was 59.05 ± 1.21 years, respectively. There was no significant difference in physical and serum biochemical parameters between the sham group and the LiESWT group. The biochemical parameter-related diabetes mellitus, liver or renal function, and hyperlipidemia were also recorded: All participants were within normal range, and none had metabolic syndrome-related OAB.

#### 2.2.2. Primary and Secondary End Points

**Figure 5:**
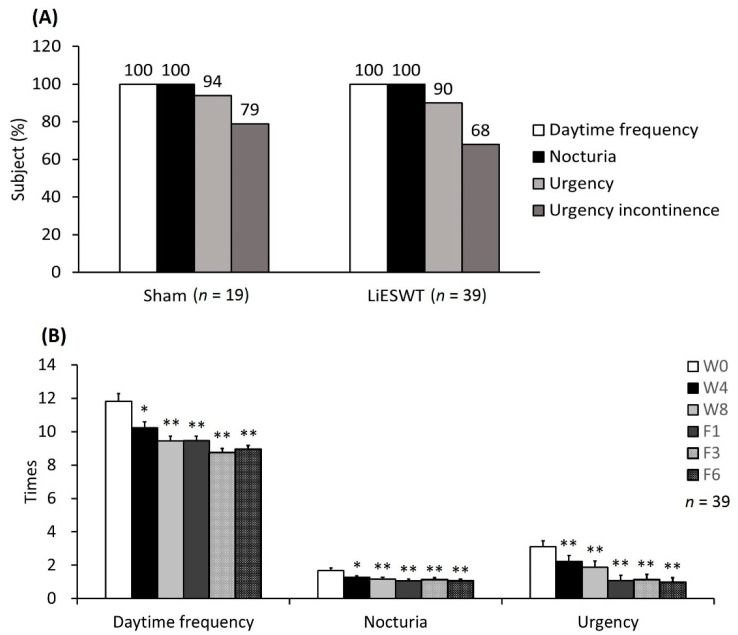
Analysis of postmenopausal participants with OAB symptoms. (**A**) The percentage of postmenopausal participants with OAB symptoms. OAB consisted of daytime frequency, nocturia urgency with or without urgency incontinence. (**B**) The therapeutic efficacy of LiESWT improved the OAB symptoms evaluated by 3-day urinary diary. The changes in daytime frequency, nocturia and urgency by 3-day urinary diary record at W4, W8, F1, F3, and F6 compared with W0. The mean times of daytime frequency, nocturia and urgency were noticeably decreased after LiESWT treatment. W0: the baseline, W4: 4**-**week of LiESWT treatment, W8: 8**-**week of LiESWT treatment, F1: 1-month follow-up, F3: 3-month follow-up, F6: 6-month follow-up. * *p* < 0.05; ** *p* < 0.01 as compared with W0.

The percentages of postmenopausal participants with OAB with daytime frequency and nocturia were 100% in both groups. Besides, the proportion of urgency ≥ 1 time and urgency incontinence ≥ 1 time were 94% and 79% in the sham group, respectively. In the LiESWT group, the percentages of urgency and urgency incontinence were 90% and 68% (Figure 5A). From the results of 3-day urinary diary, LiESWT meaningfully decreased daytime frequency, nocturia, and urgency of OAB symptoms as early as 4 weeks of treatment (W4), and the effect was more significant at 8 weeks (W8), while persisting after 1, 3, and 6 months of follow-up (F1, F3, and F6). Data are shown in Figure 5B and Table 3.

In comparison with W0, there was no significant difference in the mean values of fluid intake, urine output, average voided volume, FBC, daytime frequency, nocturia, or urgency times in the sham group after 4 weeks of treatment (W4). However, the mean values of average voided volume in the LiESWT group were noticeably increased from W0 (186.8 ± 7.6) to W8 (213.2 ± 6.5, *p* < 0.01), F1 (213.6 ± 7.1, *p* < 0.01), F3 (218.4 ± 7.9, *p* < 0.01), and F6 (209.0 ± 7.6, *p* < 0.05), respectively. The mean values of FBC in the LiESWT group were obviously increased from W0 (338.1 ± 11.2) to W8 (376.0 ± 11.8, *p* < 0.01), F1 (378.9 ± 14.8, *p* < 0.01) and F3 (356.7 ± 11.5, *p* < 0.05). Taken together, LiESWT improved the OAB symptoms by decreasing daytime frequency, nocturia, and urgency as early as 4 weeks and more prominent after 8 weeks of treatment. Such therapeutic effects could last after 6 months of follow-up.

The bladder storage and void function were objectively evaluated by uroflowmetry (voided urine volume and Qmax) and PVR (Table 3). In comparison with W0, there was no significant difference in the voided urine volume, Qmax, and PVR in W4 of the sham group. However, the mean value of voided urine volume (ml) in the LiESWT group was noticeably increased from W0 (314.8 ± 14.6) to W4 (369.6 ± 14.1, *p* < 0.01), W8 (392.5 ± 17.6, *p* < 0.01), F1 (380.7 ± 14.0, *p* < 0.01), F3 (362.8 ± 14.2, *p* < 0.01), and F6 (359.7 ± 13.1, *p* < 0.05). The Qmax (mL/s) in the LiESWT group was significantly increased from W0 (24.21 ± 1.09) to W4 (27.58 ± 14.3, *p* < 0.05), W8 (28.35 ± 1.15, *p* < 0.05), F1 (28.09 ± 1.39, *p* < 0.05), F3 (26.98 ± 0.90, *p* < 0.05), and F6 (26.98 ± 1.14, *p* < 0.05). Besides, PVR (ml) in the LiESWT group also exhibited a significant decrease from W0 (46.67 ± 5.27) to W4 (35.06 ± 4.63, *p* < 0.05), W8 (31.01 ± 4.94, *p* < 0.01), F1 (21.45 ± 1.97, *p* < 0.01), F3 (26.62 ± 2.97, *p* < 0.01), and F6 (24.16 ± 2.92, *p* < 0.01), respectively. Taken together, voided urine volume, Qmax, and PVR were noticeably improved at W4, W8, F1, F3, and F6 as compared with W0 in the LiESWT group. These results implied bladder voiding efficacy improved as early as 4 weeks of LiESWT treatment. Such beneficial efficacy of LiESWT could be maintained up to six months of follow-up.

**Figure 6:**
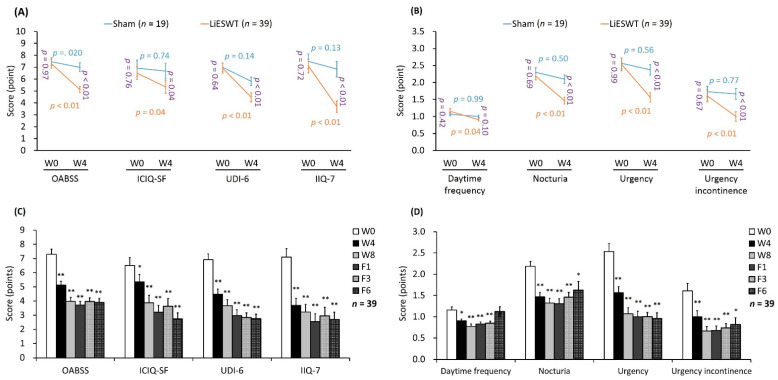
Improvement in OAB symptoms and life bothersome questionnaire scores after LiESWT treatment. (**A**,**C**) The therapeutic effect of LiESWT was analyzed by the OAB symptoms and life bothersome questionnaires, including OABSS, ICIQ**-**SF, UDI**-**6, and IIQ**-**7. LiESWT treatment significantly reduced in the scores of OABSS, ICIQ**-**SF, UDI**-**6, and IIQ**-**7 as compared with the sham group. (**B**,**D**) Improvement of OABSS sub-scores for OAB symptoms after LiESWT treatment, including daytime frequency, nocturia, urgency, and urgency incontinence. LiESWT improved OAB symptoms and the QoL. Note: OABSS, overactive bladder symptom scores. ICIQ**-**SF: international consultation on incontinence questionnaire**-**short form, UDI**-**6: urogenital distress inventory**-**6, IIQ**-**7: incontinence impact questionnaire**-**7, W0: the baseline, W4: 4 weeks of LiESWT treatment, W8: 8 weeks of LiESWT treatment, F1: 1**-**month follow-up, F3: 3**-**month follow-up. The blue or orange font denotes the *p*-value before and after 4 weeks treatment in the sham group or in the LiESWT-treated group, respectively. The purple font indicates the *p*-value between the sham group and the LiESWT-treated group at the W0 and W4. * *p* < 0.05; ** *p* < 0.01 as compared with W0.

The therapeutic effects of LiESWT on social activity and QoL were investigated by the OAB symptom scores and life bothersome questionnaire scores, including OABSS, ICIQ-SF, UDI-6, and IIQ-7 (Table 3 and Figure 6). In comparison with W0 data, there was no meaningful difference in the scores of OABSS (*p* = 0.20), ICIQ-SF (*p* = 0.74), UDI-6 (*p* = 0.14), and IIQ-7 (*p* = 0.13) in W4 of the sham group (Figure 6A). However, in W4 of the LiESWT group, the scores of OABSS (*p* < 0.01), ICIQ-SF (*p* < 0.05), UDI-6 (*p* < 0.01), and IIQ-7 (*p* < 0.01) were significantly declined as compared with W4 of the sham group (Figure 6C). According to life bothersome questionnaire, the scores of OABSS, ICIQ-SF, UDI-6, and IIQ-7 were meaningfully decreased at W4, W8, F1, F3, and F6 as compared to W0. These findings indicated that LiESWT caused obvious improvements in OAB symptoms and QoL.

OABSS score was used for objective and quantitative assessment of OAB symptoms. According to the four sub-scores of OABSS (Table 3 and Figure 6B), there was no meaningful difference in the daytime frequency (*p* = 0.99), nocturia (*p* = 0.50), urgency (*p* = 0.56), and urgency incontinence (*p* = 0.77) in W4 as compared with W0 of the sham group (Figure 6B). Furthermore, LiESWT noticeably decreased the mean times of daytime frequency (1.22 ± 0.07 vs. 0.86 ± 0.04, *p* < 0.05), nocturia (2.19 ± 0.11 vs. 1.47 ± 0.10, *p* < 0.01), urgency (2.53 ± 0.19 vs. 1.57 ± 0.14, *p* < 0.01), and urgency incontinence (1.61 ± 0.18 vs. 1.00 ± 0.14, *p* < 0.01) as W0 versus W4, respectively. The mean of daytime frequency in the LiESWT group was significantly increased from W0 (1.22 ± 0.07) to W4 (0.86 ± 0.04, *p* < 0.05), W8 (0.77 ± 0.05, *p* < 0.01), F1 (0.83 ± 0.05, *p* < 0.01), F3 (0.85 ± 0.05, *p* < 0.01), and F6 (1.13 ± 0.11). The mean value of nocturia in the LiESWT group was noticeably increased from W0 (2.19 ± 0.11) to W4 (1.47 ± 0.10, *p* < 0.01), W8 (1.32 ± 0.11, *p* < 0.01), F1 (1.31 ± 0.11, *p* < 0.01), F3 (1.46 ± 0.11, *p* < 0.01), and F6 (1.63 ± 0.20, *p* < 0.05). Similar treatment effects also occurred in urgency and urgency incontinence in the LiESWT group. Such effect sustained until 6 months of follow-up (Table 3, Figure 6B,D).

#### 2.2.3. Safety of LiESWT Treatment

For safety considerations, LiESWT was well tolerated by all participants. There were no adverse effects observed, neither skin ecchymosis, intolerable pain, nor hematuria occurred in the present study.

#### 2.2.4. A Proposed Diagram for the Therapeutic Effect of LiESWT on Altering Bladder Angiogenesis and Improving OHD-Induced OAB

**Figure 7:**
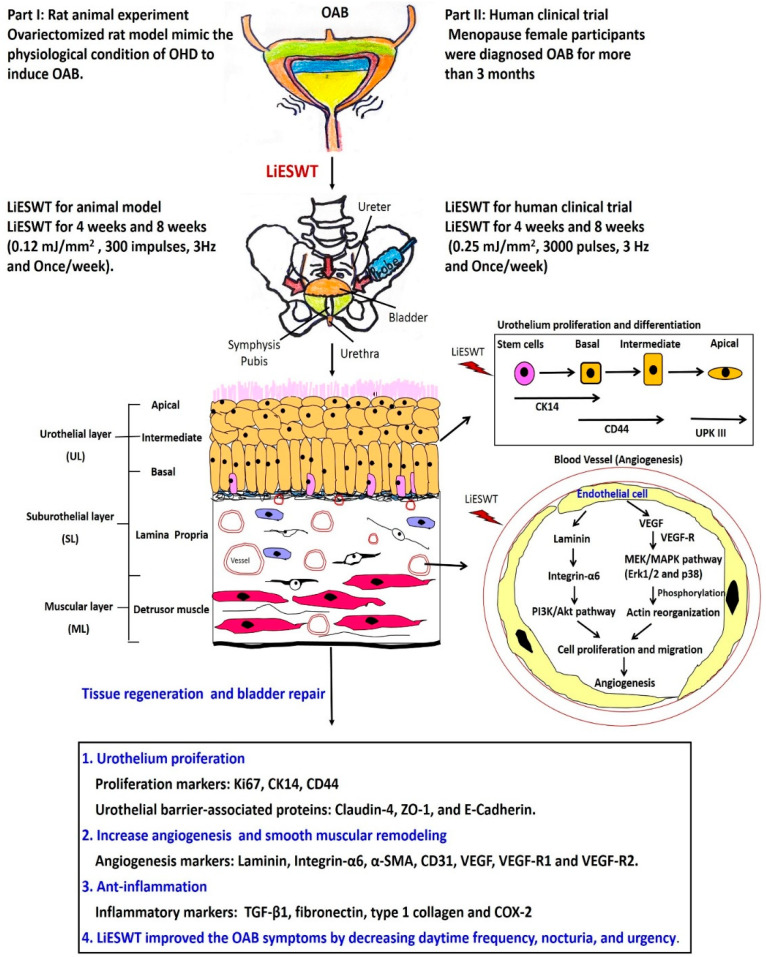
A study diagram proposed for the potential effect of LiESWT on improving the OAB symptoms. Part I: Rat animal experiment: LiESWT (0.12 mJ/mm^2^, 300 impulses, 3 Hz, and once/week) for OVX-induced OAB rat was placed on the lower abdomen. The effect of LiESWT enhanced anti-inflammation, promoted cell proliferation, and altered angiogenesis through VEGF/VEGF**-**R/MAPK (P38 and Erk1/2) pathways and Laminin/Integrin**-**α6/Akt pathways to improve OAB-induced by OHD. Part II: Human clinical trial: LiESWT (0.25 mJ/mm^2^, 3000 pulses, 3 Hz, and once/week) for postmenopausal OAB participants was placed on the lower abdomen with two fingers apart from the pubis. The results revealed that LiESWT improved bladder function and ameliorated the OAB symptoms after 8**-**week LiESWT. Note: OVX, ovariectomy; OHD, ovary hormone deficiency; OAB, overactive bladder; LiESWT, low-intensity extracorporeal shock wave therapy; CK, cytokeratin; VEGF, vascular endothelial growth factor; TGF**-**ß1, transforming growth factor ß 1.

According to the above results, a brief diagram was proposed for the therapeutic effect of LiESWT on OHD-induced OAB in a human clinical trial and rat model (Figure 7). The OVX rat model mimicked the physiological condition of OHD and thereafter induced OAB, including urinary frequency and involuntary detrusor contraction. The effect of LiESWT enhanced anti-inflammation, promoted cell proliferation, and altered angiogenesis through the VEGF/VEGF-R/MAPK (P38 and Erk1/2) pathway and Laminin/Integrin-α6/Akt pathway to ameliorate OHD-induced OAB (Figure 7).

In part II of the human clinical trial, postmenopausal participants aged 40–75 years who were diagnosed of OAB for more than 3 months were enrolled. The effect of LiESWT (0.25 mJ/mm^2^, 3000 pulses, 3 Hz, and once/week) on OAB was analyzed by 3-day urinary diary, uroflowmetry, PVR, and life bothersome questionnaires. The results revealed that LiESWT improved bladder function and ameliorated the OAB symptoms, including urinary incontinence, urgency, frequency, and nocturia after 4- and 8-weeks of LiESWT treatment. Such effects greatly promoted social activity and QoL (Figure 7).

## 3. Discussion

The OAB animal model results revealed that OVX obviously caused urothelial atrophy, increased interstitial fibrosis, and inhibited angiogenesis. Treatment with LiESWT significantly reduced micturition pressure as well as micturition frequency, and increased bladder capacity as compared with the OVX group. Moreover, OVX significantly exacerbated bladder mucosa atrophy and enhanced interstitial fibrosis. In contrast, LiESWT treatment reduced the fibrotic biosynthesis and ameliorated the bladder damage. LiESWT also increased bladder regeneration, induced angiogenesis, and increased the phosphorylation of P38, Erk1/2, and Akt, but reduced the phosphorylation of C-Jun. In human clinical trial, LiESWT noticeably increased the mean voided urine volume, FBC, and Qmax, while the PVR volume was significantly declined. According to life bothersome questionnaires, the scores were significantly decreased at W4, W8, F1, F3, and F6 as compared to baseline. The therapeutic efficacy of LiESWT not only exhibited significant improvement in FBC, average voided volume, and Qmax, but also ameliorated OAB symptoms, including frequency, nocturia, and urgency incontinence, and at last improved patients’ social activity and QoL. To sum up, the molecular mechanism underlying the therapeutic effect of LiESWT on OAB might be through ameliorating bladder overactivity, inhibiting interstitial fibrosis, promoting cell proliferation, enhancing angiogenesis, and elevating the phosphorylation of ErK1/2, P38, and Akt.

Clinical application of LiESWT for various urological disorders, such as ED [32,33,34,35,36], IC/BPS [40], diabetes-induced underactive bladder [42,43], OAB [38], and SUI [39], have been recently explored. Gruenwald et al. implied the efficacy of LiESWT in severe ED patients who respond poorly to PDE5i therapy and suggested that LiESWT significantly improved erectile rigidity and sexual satisfactions [44]. ED patients treated with LiESWT (0.10–0.25 mJ/mm^2^ and 3000–6000 pulses once per week for 4–8 weeks) promoted penile tissue regeneration and penile neovascularization [32,33,34,44]. The data suggested that LiESWT significantly improved perineum pain and micturition dysfunction, erectile function, and QoL as compared with the sham group [27,28,29]. Besides, Zimmermann et al. investigated the effect of LiESWT (0.10–0.25 mJ/mm^2^, 3000 impulses for 4 weeks) on CPPS-significantly improved pain and micturition, as well as erectile function and QoL [27,45]. Our previous clinical investigation wherein we performed the therapeutic effect of LiESWT on female SUI revealed that 8-week LiESWT (0.25 mJ/mm^2^ intensity, 3000 pulses, 3 Hz, and once weekly) could not only noticeably improve stress urine leakage, but also ameliorate urinary frequency, nocturia, and urgency. Such therapeutic effects improved social activity as well as QoL and persisted after 1 month of follow-up [39]. Our results also revealed that 8-week LiESWT (0.25 mJ/mm^2^ and 3000 pulses) meaningfully improved average urine volume, FBC, Qmax, PVR, and attenuated OAB symptoms. Such effects were significantly improved and could last to six months.

As is well known, the occurrence of OAB is correlated with estrogen deficiency. Postmenopausal women with estrogen deficiency have LUTS-related urogenital atrophy [18,46]. Estrogen especially estradiol is essential for mediating physiologic functions in the female bladder [9]. The effects of estrogen were generated via estrogen receptors (ERs), including ER-α and ER-β, shown in the lower urinary tract, including bladder trigone, proximal urethra, vagina, and vesicovaginal connective tissue contiguous with the bladder neck [47]. Previous studies implied that the rat model with estrogen-depletion lacked the urothelial mucosal barrier, damaged mucosal integrity, and changed the prostaglandin level [15]. The decreasing estrogen level caused atrophic changes in vulvar, vaginal, urethral, and bladder tissue [26]. In animal studies, estradiol suppressed the expression of rho-kinase and declined detrusor smooth muscle contraction [48]. Dobberfuhl et al. implied that estradiol supplement in OVX rabbits could reduce oxidative stress on bladder tissue, improve the harmful effect of hypoxia on the pelvic floor, and restore bladder contractile function [49]. These results, in accordance with our study, implied that the OVX rat model for one year can be applied for mimicking women postmenopausal status with OHD to induce OAB with an increase in micturition frequency and bladder storage dysfunction [14,19]. The morphological results also revealed that the OVX group obviously caused urothelial atrophy, increased interstitial fibrosis, and inhibited angiogenesis. In contrast, animal application of LiESWT could increase bladder urothelium regeneration, induce angiogenesis, and increase the phosphorylation of P38, Erk1/2, and Akt. Besides, clinical application of LiESWT in postmenopausal OAB participants not only enhanced the bladder voiding function and ameliorated OAB symptoms, but also improved social activity and QoL.

Lower urinary tract dysfunctions in postmenopausal women have been linked to pelvic organ ischemia and hypoxia [49]. Angiogenesis might be a vital role in maintaining blood nutrition and oxygen to supply bladder regeneration. The mechanism of angiogenesis by the VEGF-signaling pathway was through VEGF receptor and was involved in stimulating phosphorylation of Erk1/2, P38, and Akt [50]. VEGF protein has been found to be reduced in chronic ketamine abusers with bladder overactivity [51]. Zhu et al. showed that a combination of mesenchymal stem cell (MSC) treatment and LiESWT could induce vasodilatation and angiogenesis, while the effect was through the activation of PI3K/Akt/mTOR signal pathways and NO/cGMP signal pathways [52]. This combination therapy could provide a new treatment strategy in ED patients [53]. Additionally, LiESWT promoted angiogenesis to enhance tissue repair via mediators such as VEGF, endothelial nitric-oxide synthase (eNOS), hypoxia-inducible factor 1a, and CD31 [54,55,56]. The effect of LiESWT was involved in modulating fibroblast recruitment, decreasing the production of oxygen radicals, attenuating leukocyte infiltration and suppressing tissue apoptosis to increase tissue remodeling [57]. Following LiESWT treatment, enhanced VEGF-R2/Akt/NOS signaling pathways and cellular NO production, increased cellular migration, and enhanced microvessel formation in ischemic limb in obese mice have been reported [58]. In primary human adipose tissue-derived stem cells and murine mesenchymal progenitor cells, LiESWT treatment promoted cell proliferation, ATP release, and Erk1/2 and p38 MAPK pathway activation [59]. In addition, LiESWT promoted myogenesis through the protein kinase RNA-like ER kinase (PERK)/eukaryotic initiation factor-2a (eIF-2α)/activating transcription factor 4 (ATF4) signaling pathway. Besides, an PERK inhibitor (GSK2656157) effectively inhibited the myotube formation in rat myoblast cells and attenuated myotube formation induced by LiESWT [60]. Furthermore, LiESWT triggered the release of cellular ATP to activate purinergic receptors via Erk activation and finally enhanced cell proliferation to improve wound healing [59]. Rat experiments also revealed that LiESWT (0.10–0.13 mJ/mm^2^ and 200–300 impulses) improved bladder, induced angiogenesis, and inhibited oxidative stress in rats with CYP-induced IC [41,61]. Moreover, LiESWT could enhance endothelial NO synthase (eNOS) activity, which results in the suppression of NF-κB to decrease inflammation [62].

Based on our findings, the level of angiogenesis related proteins and receptors (laminin, integrin-α6, α-SMA, CD31, VEGF, VEGF-R1, VEGF-R2) in the OVX group was significantly suppressed compared to the sham group and the OVX + SW group (Figure 4). Moreover, LiESWT treatment significantly promoted the phosphorylation of Erk1/2, P38, and Akt in the bladder of the OVX + SW4 group and the OVX + SW8 group as compared with the OVX group, whereas it reduced the phosphorylation of C-Jun. LiESWT induced angiogenesis and activated the phosphorylation of P38, Erk1/2, and Akt, but reduced the phosphorylation of C-Jun**.** From our perspective, LiESWT promoted bladder angiogenesis through the VEGF/VEGFR/MAPK (P38 and Erk1/2) pathway and Laminin/Integrin**-**α6/Akt pathway by increasing capillary density to improve OHD-induced OAB.

In view of morphological evaluations and Western blots results, cell proliferation and differentiation proteins (Ki67, CD44, CK14, and Uroplakin III), urothelial tight junction-associated proteins and adhesion molecules (Claudin-4, ZO-1, and E-Cadherin), and angiogenesis-related proteins and receptors (laminin, integrin-α6, α-SMA, CD31, VEGF, VEGF-R1, VEGF-R2) in the OVX + SW8 group were significantly enhanced in comparison with the OVX group. Our results indicated that the OVX group significantly increased urothelial atrophy, suppressed angiogenesis, and exacerbated bladder pathological damage. Nevertheless, LiESWT could promote bladder repair through urothelial regeneration, differentiation, and increasing CD44 (HA receptor) expression. In particular, there are many mononuclear cells gathered into the spherical shape and collagen accumulation around the mononuclear cell sphere in the SL of the OVX + SW8 group. The immunostaining expression of proliferating markers Ki67 and CK14 were obviously displayed in UBL and the sphere of SL in the OVX + SW8 group compared to the OVX group. Besides, the protein levels of proliferation and urothelial tight junction proteins were obviously enhanced in the OVX + SW8 group compared to the OVX group and the OVX + SW4 group. These findings demonstrated LiESWT could elevate fibroblast recruitment, increase cell proliferation, differentiation, and increase HA expression to improve bladder repair. Previous studies have implicated that HA was involved in wound healing and tissue regeneration, and endothelial cell proliferation, as well as angiogenesis. HA interacted with HA receptors, including the cluster of CD44, receptor for HA-mediated motility (RHAMM), and Toll-like receptor-4 (TLR-4) to regulate urothelial cell proliferation, differentiation, and migration [63,64]. Transgenic mice with the expression of an antisense CD44 cDNA under the control of the keratin-5 promoter exhibited defective keratinocyte proliferation, indicating that HA receptor CD44 played an important role in the regulation of keratinocyte proliferation in response to extracellular stimuli [65].

Considering participant safety and interference factors, we have exclusion criteria in Table 4. We examined physical indicators (height, weight, waistline, body mass index, and blood pressure) and the lipid profile (triglycerides, cholesterol, low-density lipoprotein, and high-density lipoprotein) in Table 2 to rule out metabolic syndrome and abdominal fat limitation. The transmission process of the shockwave machine by the probe needed mediator-distilled water. The probe was placed on the low abdominal skin with the ultrasound transmission gel over the bladder dome and bilateral wall to avoid air interference. All participants were asked to drink water, then received LiESWT treatment after filling the bladder to 50% FBC by using bladder scan sonography. In particular, before LiESWT in the rat model, the rat’s lower abdomen had to be shaved so as not to interfere with the effect of LiESWT. The probe was gently placed on the ultrasound transmission gel overlying the area of the bladder. During LiESWT treatment, some participants felt a slight tingling sensation and some had no sensation. However, in this study, there were no adverse effects occurred, such as intolerable pain, gross hematuria, or skin ecchymosis.

The present study provided the potential therapeutic mechanism of LiESWT on OHD-induced OAB. Animal models are an important research tool for investigating the molecular mechanism of OAB. The underlying molecular mechanism of anti-inflammation, proliferation, angiogenesis, and neurogenesis of LiESWT on OAB patients should be evaluated in the future. Unfortunately, owing to the fact that OAB is a symptom-based diagnosis, development of an animal model of the condition is hindered by the lack of a corroborating biomarker-specific for OAB to predict treatment outcome.

The present study was designed as a prospective, randomized, single-blinded, parallel clinical trial to investigate the efficacy of LiESWT on postmenopausal OAB patients. However, there were several limitations in the present study. First, some participants of the sham group withdrew due to a lack of obvious improvement after sham treatment. Therefore, we only had W4 data in the sham group and lack of W8, and follow-up (F1, F3, and F6). The number of participants in the sham group (19 subjects) was also different from the LiESWT group (39 subjects). Consequently, two quantitative statistical methods were analyzed. The intergroup (sham group vs. LiESWT) relationships of W0 and W4 data were evaluated by using the Student’s *t*-test. Moreover, a paired *t*-test was used to perform a repeated measurement analysis for the intragroup before/after treatment and to calculate *p*-values for comparison. Second, the optimal protocol for LiESWT treatment for OAB patients remained unclear. Different treatment intervals (once or twice a week) and different frequencies and energy delivered must be further evaluated in the future to determine the optimal protocol for OAB. Third, the therapeutic efficacy of LiESWT on male OAB participants require future exploration. Fourth, urgency is a key symptom of overactive bladder. However, it is difficult to define urgency clearly and some participants might have ignored it and not record it in our questionnaire or voiding diary.

In conclusion, LiESWT attenuated inflammatory responses, increased angiogenesis, promoted proliferation, and differentiation reduced urinary leakage and improved OAB symptoms, thereafter promoting the quality of life.

## 4. Materials and Methods

### 4.1. Part I: Animal experiment

#### 4.1.1. Experimental Design of Animal Model and LiESWT Protocol

Female Sprague–Dawley rats weighing between 200 and 250 g were purchased from the animal center of Biolasco Taiwan Co., Ltd. Taipei, Taiwan. The experimental procedures were approved by the Committee for the Use of Experimental Animal of Kaohsiung Medical University and adhered to the guidelines of the National Institute of Health. Thirty-six female rats were divided into four groups, including (1) the sham group, (2) the OVX group: ovarian hormone deficiency (OHD) induced by the bilateral OVX surgery for 12 months, (3) the OVX + SW4 group: LiESWT once weekly for 4 weeks following 12-month OVX, and (4) the OVX + SW8 group: LiESWT once weekly for 8 weeks following 12-month OVX [66,67]. OVX surgery was performed under halothane anesthesia. The skin of female rats was cut in parallel bilateral sides of the spine about 1 cm, and both ovaries were excised to induce OHD. After OVX operation for two weeks, we checked serum estradiol concentration. Symptoms of OAB, including urinary frequency and involuntary detrusor contraction were recorded by cystometry and physiological metabolic cage after 12-month-OVX thereafter.

#### 4.1.2. LiESWT Treatment for Animal Model

The animals were anesthetized with isoflurane. After shaving the abdominal skin, the DUOLITH SD1-TOP-focused shock wave system (STORZ MEDICAL, AG, Kreuzlingen, Switzerland) applicator was gently placed with ultrasound transmission gel overlying the area of the bladder, then tilted to 45°, proceeding in a clockwise direction around the whole bladder to make sure that different parts of the bladder had received LiESWT energy transmission with 300 impulse shock wave numbers, 0.12 mJ/mm^2^ intensity, and 3 pulses/second frequency once a week for 4 or 8 weeks as modified from previous reports [61,68].

#### 4.1.3. Evaluation of Estradiol Level

Blood samples were collected to evaluate serum 17β estradiol concentration after bilateral OVX for 12 months. Blood was separated by centrifugation at 4 °C. According the 17β estradiol ELISA kit (Cayman Chemical Co., Ann Arbor, MI, USA) manufacturer’s protocol, the standard and serum samples were added to microtiter wells which were coated with anti- estradiol primary antibody and incubated for 2 h at room temperature. After the addition of the 100-L substrate solution and incubation for 15 min at room temperature, the 50 μL of stop solution was added and the concentration of estradiol was measured at 450 nm by the ELISA reader (Bio-Tek ELX 800, BioTek, Bad Friedrichshall, Germany) [13]. The estradiol level of rat serum was determined using a reference standard curve.

#### 4.1.4. Isovolumetric Cystometrograms (CMGs)

The CMG was measured in accordance with the method previously described [13,14]. The rats were anesthetized by injection of Zoletil-50 (1 mg/kg, IP). The PE-50 tubing was inserted into the bladder through the urethra. The tubing was connected via a 3-way stopcock to a pressure transducer and to a syringe pump to record intravesical pressure. Subsequently, the bladder was infused with 0.9% saline at a steady rate (0.08 mL/min) to measure the bladder pressure. The bladder pressure was recorded at least 5 filling/voiding stable cycles on each rat. Pressure and force signals were amplified (ML866 PowerLab, ADInstrument, Colorado Springs, CO, USA), recorded on a chart recorder, and digitized for computer data collection (Labchart 7, ADInstruments: Windows 7 system) [69,70]. CMG parameters recorded for each rat included filling pressure, peak micturition pressure, bladder capacity, and the frequency of voiding and non-voiding contractions (without urine leakage during bladder infusion).

#### 4.1.5. Physiological Metabolic Cage for Tracing Voiding Behavior Studies

The rats were placed in individual KDS-TL380 metabolic cages (R-2100; Lab Products, Rockville, MD, USA). The 24-h micturition frequency and voided volume were determined using the MLT0380 transducer (MLT 0380, ADI Instruments, Colorado Springs, CO, USA). The volume of water intake and urine amount were collected and measured. The volume of liquid consumed, 24-h micturition frequency, and 24-h urine volume were recorded for 3 days and an average value was determined [69,70].

#### 4.1.6. Histological Study by Masson’s Trichrome Stain

The whole bladders were removed to record weight, and they were cut open in a sagittal direction. To analyze the whole bladder morphology, the bladder tissues from different groups were embedded in paraffin blocks and 5-μm-thick sections were obtained. Deparaffinized sections were stained with Masson’s trichrome stain (Masson’s Trichrome Stain Kit, DAKO, Santa Clara, CA, USA). The navy blue-stained nucleus, green-stained collagen, and red-stained smooth muscle were highlighted for each image [13]. The sections were captured by digital camera in ten random, non-overlapping frames at 400X magnification, and the entire bladder wall thickness. The color setting and image-associated quantification were determined by image analysis software (Image-Pro Plus, Media Cybernetics, Rockville, MD, USA).

#### 4.1.7. Protein Isolation and Western Blot Analysis

Frozen bladder tissues were homogenized on ice in buffer (50 mM Tris (pH 7.5) and 5% Triton X-100) containing halt protease inhibitor cocktail (Pierce, Rockford, IL, USA) at 100 mg/mL. Then, 16 μg of protein extracted from the bladders was loaded onto SDS-PAGE gels and transferred to polyvinylidene fluoride membranes (PVDF membrane, Millipore, Burlington, MA, USA). The membranes were incubated with the primary antibodies for transforming growth factor-β1 (TGF-β1) (Bioss, Woburn, MA, USA, rabbit polyclonal IgG 1:1000, MW: 44 kDa, catalog no. bs-0086R), fibronectin (Merck, Kenilworth, NJ, USA, mouse monoclonal IgG1, 1:1000, MW: 262 kDa, catalog no. MAB 1940), type 1 collagen (Abcam, Cambridge, UK, 1:1000, rabbit polyclonal IgG, MW: 139 kDa, catalog no. ab34710), cyclooxygenase-2 (COX-2) (Cayman, Ann Arbor, MI, USA, mouse monoclonal IgG, 1:1000, MW: 74 kDa, catalog no. 160126), uroplakin III (UPK III) (Abcam, mouse monoclonal IgG1, 1:1000, MW: 32 kDa, catalog no. ab78196), E-Cadherin (Proteintech, Rosemont, IL, USA, rabbit polyclonal IgG 1:1000, MW: 97 kDa, catalog no. 20874-1-AP), Ki67 (Abcam, rabbit monoclonal IgG 1:1000, MW: 358 kDa, catalog no. ab1667), CK14 (GeneTex, Irvine, CA, USA, rabbit polyclonal IgG, 1:1000, MW: 52 kDa, catalog no. GTX104124), CD44 (Cell Signaling, Danvers, MA, USA, mouse monoclonal IgG1, 1:1000, MW: 80 kDa, catalog no. #5640), claudin-4 (Invitrogen, Waltham, MA, USA, mouse monoclonal IgG, 1:1000, MW 22 kDa, catalog no. 329400), zona occludens (ZO-1) (Invitrogen, rabbit monoclonal IgG, 1:1000, MW 220 kDa, catalog no. 40-2200), laminin (Abcam, rabbit polyclonal IgG, 1:1000, MW: 200~400 kDa, catalog no. ab7463), integrin-α6 (Abcam, rabbit monoclonal IgG, 1:5000, MW: ~127 kDa, catalog no. ab181551), alpha-smooth muscle actin (α-SMA) (Abcam, rabbit polyclonal IgG, 1:6000, MW: 40, 42 kDa, catalog no. ab5694), CD31 (Abcam, mouse monoclonal IgG, 1:3000, MW: 83 kDa, catalog no. 9498), vascular endothelial growth factor (VEGF) (Merck, mouse monoclonal IgG, 1:1000, MW: ~40 kDa, catalog no. MABC595), vascular endothelial growth factor-receptor 1 (VEGF-R1) (Abcam, rabbit monoclonal IgG, 1:1000, MW: ~150 kDa, catalog no. ab32152), endothelial growth factor-receptor 2 (VEGF-R2) (Cell Signaling, rabbit monoclonal IgG, 1:1000, MW: ~220 kDa, catalog no. #9698), Erk1/2 (p44/42; Cell Signaling, rabbit polyclonal IgG, 1:6000, MW: 42~44 kDa, catalog no. #9102S), Phospho-Erk1/2 (p-Erk1/2; Cell Signaling, rabbit monoclonal IgG, 1:1000, MW: 42~44 kDa, catalog no. #4370S), P38 (Cell Signaling, rabbit monoclonal IgG, 1:6000, MW: ~40 kDa, catalog no. #8690), Phospho-P38 (p-P38; Cell Signaling, rabbit monoclonal IgG, 1:1000, MW: ~40 kDa, catalog no. #4511), C-Jun (Biocompare, San Francisco, CA, USA, rabbit monoclonal IgG, 1:1000, MW: ~48 kDa, catalog no. #9165S), Phospho-C-Jun (p-C-Jun; Cell Signaling, rabbit monoclonal IgG, 1:1000, MW: ~48 kDa, catalog no. #3270S), Akt (Cell Signaling, mouse monoclonal IgG, 1:2000, MW: 60 kDa, catalog no. #2920), Phospho-Akt (p-Akt; Cell Signaling, rabbit monoclonal IgG, 1:1000, MW: 60 kDa, catalog no. #4060), ß-actin (Cell Signaling, rabbit monoclonal IgG, 1:5000, MW: 43 kDa, catalog no. 4970S), and glyceraldehyde-3-phosphate dehydrogenase (GAPDH) (Merck, mouse monoclonal IgG, 1:2500, MW: 36 kDa, catalog no. MAB374). Immunoreactive bands were visualized by enhanced chemiluminescence (ECL) and exposed to Biomax L film (Kodak, Rochester, NY, USA). In each experiment, negative controls without the primary antibody and protein molecular weight markers were used to exclude non-specific bands.

The inflammatory fibrosis proteins (TGF-β1, fibronectin, type 1 collagen, and COX-2), cell proliferation and differentiation proteins (Ki67, CD44, CK14, and Uroplakin III), urothelial tight junction-associated proteins and adhesion molecule (Claudin-4, ZO-1, and E-Cadherin), angiogenesis-related proteins and receptors (laminin, integrin-α6, α-SMA, CD31, VEGF, VEGF-R1, and VEGF-R2), and cell signal-related proteins (Erk1/2, p-Erk1/2, P38, p-P38, C-Jun, p-C-Jun, Akt, and p-Akt) were normalized with ß-actin and GAPDH.

#### 4.1.8. Immunofluorescence Staining for the Localization of Protein Expression

The whole bladder tissues were fixed in cold 4% paraformaldehyde in PBS (phosphate buffered saline) (0.1 M, pH 7.4) overnight, embedded in paraffin section of 5-μm thickness, and double immunofluorescence staining was performed to detect the location of target protein according to previously published methods [69,70,71]. The bladder sections were blocked with 10% NGS in PBS/0.5% Triton X-100 for 1 h and then incubated with the primary antibody to E-Cadherin (Proteintech, rabbit polyclonal IgG 1:100), Ki67 (Abcam, rabbit monoclonal IgG 1:100), CK14 (GeneTex, rabbit polyclonal IgG, 1:200), claudin-4 (Invitrogen, mouse monoclonal IgG, 1:200), and α-SMA (Abcam, rabbit polyclonal IgG, 1:100) at 4 °C overnight. Tissues were washed in PBS/0.5% Triton X-100 for 15 min and incubated with secondary antibodies (1:800; Invitrogen) at room temperature for 1 h. After PBS buffer washing, DAPI was added and cover-slipped with Prolong Gold anti-fade reagent (Invitrogen). Besides, a negative control was performed to elucidate non-specific immunostaining without the primary antibody.

#### 4.1.9. Statistical Analysis

Analysis of variance, followed by the Bonferroni test and two-way analysis of variance for individual comparison, was conducted for the above experiments. The mean, standard deviation (SD), and *p*-values were calculated on triplicate experiments. A Student’s *t*-test was used to calculate *p*-values for comparison. The significant level was set at a *p*-value < 0.05.

### 4.2. Part II: Human Clinical Trial

#### 4.2.1. Design for Human Clinical Trial

This prospective, randomized, single-blinded trial from December 2018 through January 2020 was performed in a tertiary medical center. It was carried out under the approval of Institutional Review Board (IRB No. KMUHIRB-F(II)-20180010) and was registered at clinicaltrials.gov (NCT04059133). The major inclusion and exclusion criteria are shown in Table 4. Fifty-eight postmenopausal participants who had no menstruation for at least one year and aged 40–75 years who were diagnosed with OAB symptoms (urinary urgency, frequency, nocturia, or urgency incontinence) for more than 3 months were referred for LiESWT. The postmenopausal OAB participants recruited from outpatient clinics were informed and accepted required consent, and patient history-taking and physical examination were performed. These participants were randomly assigned to two groups, including the sham (placebo) group (*n* = 19) and the LiESWT group (*n* = 39), by computer-generated random numbers. The timetable designed for the clinical trial during the LiESWT treatment procedure consisted of the baseline (W0), 4-week LiESWT (W4), 8-week LiESWT (W8), 1-month follow-up (F1), 3-month follow-up (F3), and 6-month follow-up (F6), as shown in Supplementary Figure S1 (see the Supplementary Material).

#### 4.2.2. Procedure and Medical Information of LiESWT

Postmenopausal OAB participants were informed the treatment modalities and procedures of LiESWT. The LiESWT device used was the DUOLITH SD1-TOP focused shock wave system (STORZ MEDICAL, AG). Before the LiESWT procedure, the functional bladder capacity was determined by 3-day urinary diary data and uroflowmetry data. All participants were asked to drink 1000 mL of water, and received LiESWT treatment after filling the bladder to 50% of the functional bladder capacity by using bladder scan sonography. The participants were set in the supine position, then the probe (applicator) was applied on the lower abdomen with gels applied on the skin, two fingers apart from the pubic symphysis, then tilting to 45° and targeting the bladder dome and bilateral bladder walls (each side 1000 impulses, total 3000 pulses per treatment session) at an intensity of 0.25 mJ/mm^2^, frequency of 3 pulses/second, and once a week for 4 to 8 weeks [38]. After completing the LiESWT treatment, the participants were followed up at 1 month, and then at 3 and 6 months. The probe of the placebo (sham) group was applied with air pad to block energy transmission, but the machine still emitted a shock wave generation.

#### 4.2.3. Physical and Serum Biochemical Indicators

Physical and biochemical parameters were analyzed to exclude interference factors of OAB, such as metabolic syndrome, obesity, hypertension, diabetes mellitus, and medical disease: hyperlipidemia, renal, or liver disease. Physical indicators (age, height, weight, waistline, body mass index, and blood pressure) and serum biochemical parameters, including glycated hemoglobin A1C (HbA1C), fasting blood sugar, blood urea nitrogen (BUN), creatinine, glutamate oxaloacetate transaminase (GOT; AST), glutamate pyruvate transaminase (GPT; ALT), and lipid profile (triglycerides, cholesterol, low-density lipoprotein (LDL), and high-density lipoprotein (HDL)) were analyzed to investigate the baseline characteristics of the OAB population [72]. These basal characteristics and physical as well as biochemical parameters are recorded in Table 3.

#### 4.2.4. Outcome Measurements and Therapeutic Efficacy Assessment for LiESWT

To investigate the therapeutic efficacy of LiESWT, the primary outcome parameters were performed as the change in overactive bladder symptom scores (OABSS) and 3-day urinary diary [39]. OABSS is a single symptom score to quantify OAB symptoms, including daytime frequency, nocturia, and urgency with or without urgency incontinence. Therefore, OABSS breaks down the four sub-scores. From the record of the 3-day urinary diary, urinary parameters were evaluated, such as 24-h intake and output, average voided volume, functional bladder capacity (FBC), daytime frequency, nocturia, and urinary urgency.

The secondary outcome parameters were analyzed by uroflowmetry, including voided urine volume, maximal flow rate (Qmax), and postvoid residual urinary volume (PVR). Besides, subjective satisfaction assessed with life bothersome questionnaires, such as International Consultation on Incontinence Questionnaire-short form (ICIQ-SF), Urogenital Distress Inventory-6 (UDI-6), and Incontinence Impact Questionnaire-7 (IIQ-7) at the baseline (W0), 4-week (W4), 8-week (W8), 1-month (F1), 3-month (F3), and 6-month follow-up (F6) after LIESWT treatment [38,39].

#### 4.2.5. Statistical Analysis

Statistical analyses were performed using SAS 9.3 (SAS Institute, Cary, NC, USA). Quantitative data were shown as the mean ± standard error (SE). For the intergroup comparison, Student’s *t*-test was performed. However, the paired *t*-test and one-way analysis of variance were used to perform repeated measurement analyses for intragroup before/after treatment. In order to clarify the therapeutic effect of LiESWT on postmenopausal OAB participants, the scores of pre- and post-treatment for intragroup of participants were compared. The paired *t*-test was performed in the sham group (W0 vs. W4). The post-hoc Tukey’s honestly significant difference tests were used to make comparison between the LiESWT subgroups and to calculate *p*-values for comparison. Besides, the intergroup relationship (sham group vs. LiESWT group) of W0 and W4 data were evaluated by using the Student’s *t*-test. In these analyses, *p* < 0.05 represented statistically significant [73,74].

## Figures and Tables

**Table 1:** Physical indicators and urodynamic parameters for the different experimental groups.

Variable	Sham	OVX	OVX + SW4	OVX + SW8
No. Rats	8	8	8	8
**Physical Indicators**				
Serum estradiol conc. (pg/mL) before treatment	32.17 ± 1.41	32.33 ± 1.52	31.75 ± 1.29	32.35 ± 0.53
Serum estradiol conc. (pg/mL) after treatment	33.64 ± 3.66	16.53 ± 1.28 **	15.59 ± 0.99 **	15.65 ± 1.36 **
Water intake (mL/24 h)	41.25 ± 14.36	35.50 ± 5.81	36.17 ± 5.31	32.33 ± 7.74
Urine output (mL/24 h)	20.08 ± 3.34	13.53 ± 6.78	18.12 ± 9.19	18.06 ± 7.36
Body weight (g)	400.60 ± 39.41	555.13 ± 85.95 **	524.86 ± 56.74 **	537.88 ± 75.65 **
Bladder weight (mg)	175.00 ± 19.79	163.33 ± 10.33	158.57 ± 2.37	205.00 ± 25.88 ^†^
The ratio of bladder weight (mg)/body weight (g)	0.46 ± 0.07	0.32 ± 0.02 *	0.33 ± 0.10 *	0.41 ± 0.10
**Urodynamic Parameters**				
Frequency (No. voids/1 h)	3.60 ± 0.89	6.83 ± 2.71 *	4.29 ± 1.25 ^†^	3.50 ± 0.84 ^††^
Peak micturition pressure (cm H_2_O)	25.58 ± 2.94	35.56 ± 5.73 *	25.53 ± 7.60	23.51 ± 4.41
Voided volume (mL)	2.82 ± 0.80	1.46 ± 0.36 **	2.15 ± 0.69	2.92 ± 0.65 ^††^
No. of non-voiding contractions between micturition (No. voids/h)	0.00 ± 0.00	3.17 ± 1.60 **	1.00 ± 1.73 ^††^	0.33 ± 0.52 ^††^

Footnote: OVX, surgical ovariectomy; LiESWT, low-intensity extracorporeal shockwave therapy; W4: 4 weeks of LiESWT treatment; W8: 8 weeks of LiESWT treatment; values are means ± SD. * *p* < 0.05; ** *p* < 0.01 versus the sham group. ^†^ *p* < 0.05; ^††^ *p* < 0.01 versus the OVX group.

**Table 2:** Baseline characteristics of overactive bladder (OAB) population.

Parameter	Sham	LiESWT	*p* Value	Normal Range
Physical parameter				
Female age (years)	60.79 ± 1.98	59.05 ± 1.21	0.44	
Height (cm)	159.35 ± 1.03	157.94 ± 0.80	0.30	
Weight (kg)	57.64 ± 1.83	60.69 ± 1.77	0.29	
BMI (kg/m^2^)	22.69 ± 0.66	24.28 ± 0.61	0.12	18.5–26
Waistline (cm)	81.34 ± 2.36	85.96 ± 1.88	0.15	
Systolic pressure (mmHg)	118.26 ± 3.89	123.92 ± 2.33	0.20	100–120
Diastolic pressure (mmHg)	69.74 ± 2.28	73.63 ± 1.71	0.19	60–80
MAP (mmHg)	85.91 ± 2.68	89.94 ± 1.67	0.20	70–110
Serum parameter				
HbA1C (%)	5.76 ± 0.07	5.75 ± 0.09	0.95	4–6
AC sugar (mg/dL)	103.00 ± 7.67	101.44 ± 1.81	0.61	65–109
BUN (mg/dL)	12.27 ± 0.97	13.01 ± 0.70	0.55	8–20
Creatinine (mg/dL)	0.73 ± 0.03	0.71 ± 0.02	0.65	0.44–1.03
GOT(AST) (IU/L)	23.16 ± 1.27	24.03 ± 0.83	0.56	10–42
GPT(ALT) (IU/L)	22.41 ± 2.43	23.05 ± 1.48	0.80	10–40
Triglycerides (mg/dL)	104.84 ± 10.57	111.26 ± 8.80	0.66	35–160
Cholesterol (mg/dL)	207.11 ± 7.93	208.95 ± 6.37	0.87	140–200
HDL (mg/dL)	66.14 ± 3.80	58.22 ± 2.32	0.07	29–85
LDL (mg/dL)	115.78 ± 6.62	122.76 ± 4.43	0.38	0–130

Note: BMI, body mass index; MAP, mean arterial pressure; HbA1C, hemoglobin A1c (glycated hemoglobin); AC, ante cibum (before meals); BUN, blood urea nitrogen; GOT, glutamate oxaloacetate transaminase; GPT, glutamate pyruvate transaminase; LDL, low-density lipoprotein; HDL, high-density lipoprotein; Values are means ± SE. *n* = 19 (Sham) and *n* = 39 (LiESWT).

**Table 3:** Urodynamic parameters of study population for overactive bladder (OAB).

Parameter	Sham		LiESWT					
	**W0**	**W4**	**W0**	**W4**	**W8**	**F1**	**F3**	**F6**
3-day urinary diary record								
Intake (mL)	2048.4 ± 69.4	2043.1 ± 46.1	2029.6 ± 74.6	1914.0 ± 54.4	1912.9 ± 45.5	1851.1 ± 50.8	1800.2 ± 56.3	1921.4 ± 57.2
Output (mL)	2070.9 ± 36.3	2016.4 ± 55.7	2017.4 ± 76.5	1977.1 ± 73.1	1932.6 ± 71.6	1897.9 ± 69.2	1922.9 ± 76.7	1928.8 ± 66.1
Average voided volume (mL)	186.8 ± 4.8	195.7 ± 4.5	186.8 ± 7.6	195.5 ± 6.2	213.2 ± 6.5 **	213.6 ± 7.1 **	218.4 ± 7.9 **	209.0 ± 7.6 *
FBC (mL)	344.3 ± 6.1	350.2 ± 6.4	338.1 ± 11.2	343.7 ± 11.2	376.0 ± 11.8 **	378.9 ± 14.8 **	356.7 ± 11.5 *	352.7 ± 14.7
Daytime frequency (times)	11.38 ± 0.33	11.09 ± 0.30	11.83 ± 0.46	10.24 ± 0.35 *^,†^	9.45 ± 0.28 **	9.47 ± 0.27 **	8.76 ± 0.25 **	8.96 ± 0.21 **
Nocturia (times)	1.73 ± 0.12	1.51 ± 0.11	1.68 ± 0.14	1.27 ± 0.10 *^,†^	1.17 ± 0.11 **	1.07 ± 0.10 **	1.14 ± 0.10 **	1.06 ± 0.10 **
Urgency (times)	2.90 ± 0.23	2.69 ± 0.24	3.10 ± 0.35	2.22 ± 0.36 **^,†^	1.87 ± 0.38 **	1.08 ± 0.30 **	1.14 ± 0.30 **	0.97 ± 0.26 **
Uroflowmetry data								
Voided urine volume (mL)	321.3 ± 17.6	339.0 ± 16.4	314.8 ± 14.6	369.6 ± 14.1 **	392.5 ± 17.6 **	380.7 ± 14.0 **	362.8 ± 14.2 **	359.7 ± 13.1 *
Qmax (mL/s)	25.30 ± 1.54	26.65 ± 1.18	24.21 ± 1.09	27.58 ± 1.43 *	28.35 ± 1.15 *	28.09 ± 1.39 *	26.98 ± 0.90 *	26.98 ± 1.14 *
PVR (mL)	42.79 ± 4.58	44.00 ± 4.66	46.67 ± 5.27	35.06 ± 4.63 *^,†^	31.01 ± 4.94 **	21.45 ± 1.97 **	26.62 ± 2.97 **	24.16 ± 2.92 **
OABSS score (points)								
Daytime frequency	1.07 ± 0.04	1.00 ± 0.03	1.22 ± 0.07	0.86 ± 0.04 *	0.77 ± 0.05 **	0.83 ± 0.05 **	0.85 ± 0.05 **	1.13 ± 0.11
Nocturia	2.30 ± 0.14	2.10 ± 0.13	2.19 ± 0.11	1.47 ± 0.10 **^,††^	1.32 ± 0.11 **	1.31 ± 0.11 **	1.46 ± 0.11 **	1.63 ± 0.20 *
Urgency	2.56 ± 0.16	2.38 ± 0.16	2.53 ± 0.19	1.57 ± 0.14 **^,††^	1.07 ± 0.14 **	1.00 ± 0.13 **	1.00 ± 0.10 **	0.96 ± 0.13 **
Urgency incontinence	1.73 ± 0.16	1.67 ± 0.16	1.61 ± 0.18	1.00 ± 0.14 **^,††^	0.67 ± 0.10 **	0.68 ± 0.10 **	0.74 ± 0.10 **	0.82 ± 0.15 *

Note: FBC, functional bladder capacity; Qmax, maximum flow rate; PVR, post-voided residual; SE, standard error; W, week; W4, once per week, 4-week of LiESWT; W8, once per week, 8-week of LiESWT; F1, 1-month follow-up; F3, 3-month follow-up; F6, 6-month follow-up; OABSS, overactive bladder symptom scores. Values are means ± SE. * *p* < 0.05; ** *p* < 0.01 versus W0. ^†^ *p* < 0.05; ^††^ *p* < 0.01 W4 of LiESWT versus W4 of the sham group. *n* = 19 (Sham) and *n* = 39 (LiESWT).

**Table 4:** Inclusion and exclusion criteria.

Inclusion Criteria	Exclusion Criteria
1. Menopause female participants aged 40–75 years who were diagnosed with overactive bladder (OAB) for more than 3 months.	1. Urinary tract infection detected at screening, and recurrent urinary tract infections (more than 3 episodes in the past 3 months).
2. OAB symptoms included daytime frequency of micturition ≥ 8 times, and nocturia, urgency or urgency incontinence ≥ 1 times.	2. Chronic urinary inflammation (interstitial cystitis, urethral syndrome, or painful bladder syndrome).
3. Patients could understand and follow the instructions and were able to complete the questionnaire.	3. Neuropathic diseases.
4. Patients with OAB symptom did not take antimuscarinic or ß3 agonist.	4. Lower urinary tract surgery within last 6 months.
5. OAB patient with antimuscarinic or ß3 agonist treatment could also be included after 3 months of medication withdrawal	5. Significant bladder outflow obstruction.
6. Signature of informed consent form.	6. Urinary catheterization.
	7. Drug or nondrug treatments of OAB in the previous 3 months.
	8. Perineal operations, intravesical injection, irradiation, shockwave or electrostimulation in the past 12 months.
	9. History of urolithiasis or urologic malignancy.
	10. Gross hematuria.
	11. Comorbidities associated to OAB (diabetes mellitus, spinal cord injury, stroke, or neurogenic diseases).
	12. Severe cardiopulmonary disease, liver or renal dysfunction.
	13. Previous pelvic radiation therapy.
	14. Coagulopathy.

## Data Availability

Not applicable.

## Supplementary Materials

Supplementary materials can be found online at https://www.mdpi.com/article/10.3390/ijms22179296/s1.
