# Supplementary figures and images for: Low-Intensity Extracorporeal Shock Wave Therapy Promotes Bladder Regeneration and Improves Overactive Bladder Induced by Ovarian Hormone Deficiency from Rat Animal Model to Human Clinical Trial
Source: Int J Mol Sci. 2021 Aug 27;22(17):9296. doi: 10.3390/ijms22179296 (PMC8431217; doi:10.3390/ijms22179296)

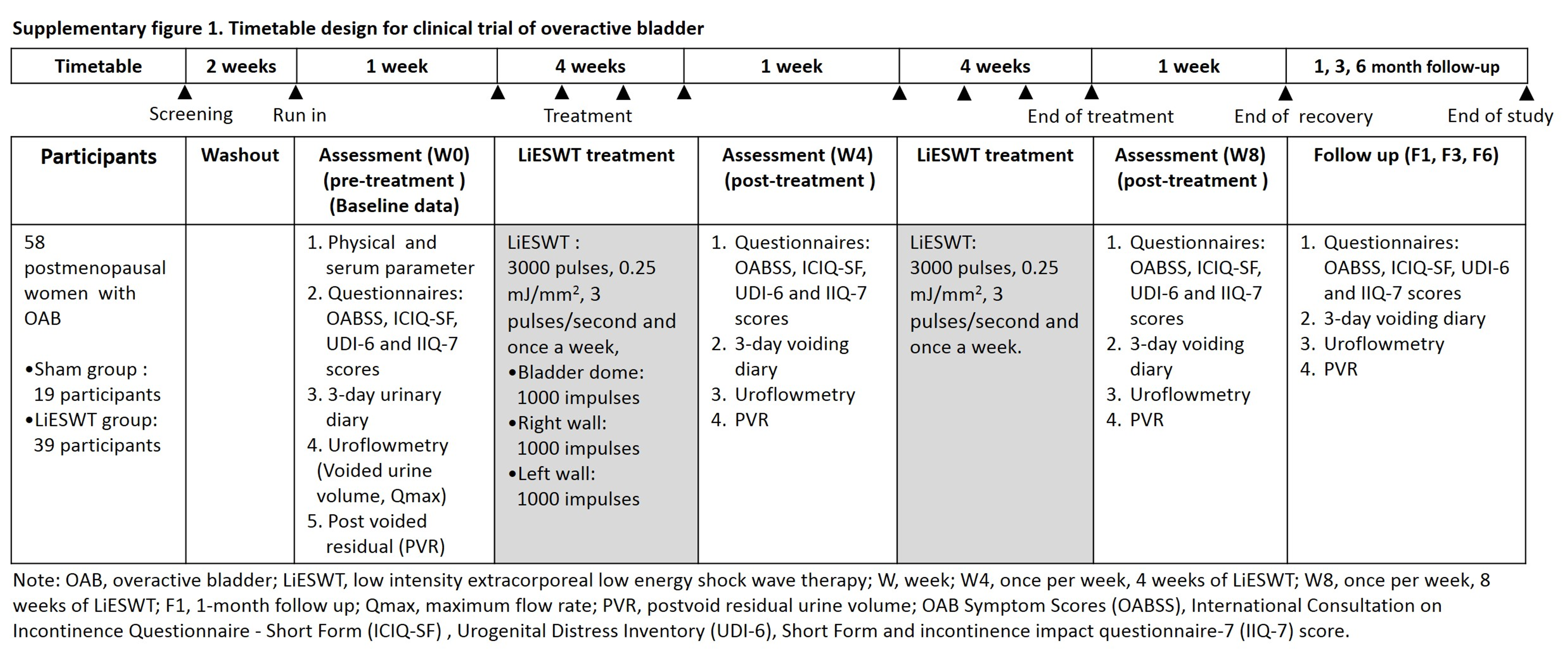

Supplement: Supplementary file 1 [file ijms-22-09296-s001.zip › ijms-1288817-supplementary.tif]
